# Keyhole Limpet Hemocyanin-Conjugated Peptides from Hepatitis C Virus Glycoproteins Elicit Neutralizing Antibodies in BALB/c Mice

**DOI:** 10.1155/2021/3108157

**Published:** 2021-01-16

**Authors:** Kai Deng, Zhanxue Xu, Mingxiao Chen, Xiaoxiang Liu

**Affiliations:** ^1^Guangzhou Eighth People's Hospital, Guangzhou Medical University, Guangzhou 510380, China; ^2^School of Pharmaceutical Sciences (Shenzhen), Sun Yat-sen University, Guangzhou 510006, China; ^3^Guangzhou Women and Children's Medical Center, Guangzhou 510623, China; ^4^Zhongshan School of Medicine, Sun Yat-sen University, Guangzhou 510600, China; ^5^Hubei Xiangyang Tuberculosis Control and Prevention Hospital, Xiangyang 441003, China

## Abstract

Currently, no vaccine to prevent hepatitis C virus (HCV) infection is available. A major challenge in developing an HCV vaccine is the high diversity of HCV sequences. The purpose of immunization with viral glycoproteins is to induce a potent and long-lasting cellular and humoral immune response. However, this strategy only achieves limited protection, and antigen selection plays a crucial role in vaccine design. In this study, we investigated the humoral immune responses induced by intraperitoneal injection of keyhole limpet hemocyanin conjugated with 4 highly conserved peptides, including amino acids [aa]317-325 from E1 and aa418-429, aa502-518, and aa685-693 from E2, or 3 peptides from hypervariable region 1 (HVR1) of E2, including the N terminus of HVR1 (N-HVR1, aa384-396), C terminus of HVR1 (C-HVR1, aa397-410), and HVR1 in BALB/c mice. The neutralizing activity against HCV genotypes 1-6 was assessed using the cell culture HCV (HCVcc) system. The results showed that the 4 conserved peptides efficiently induced antibodies with potent neutralizing activity against 3 or 4 genotypes. Antibodies induced by aa685-693 conferred potent protection (>50%) against genotypes 2, 4, and 5. Peptide N-HVR1 elicited antibodies with the most potent neutralization activities against 3 HCV genotypes: TNcc(1a), S52(3a), and ED43(4a). These findings suggested that peptides within HCV glycoproteins could serve as potent immunogens for vaccine design and development.

## 1. Introduction

Hepatitis C virus (HCV) is a leading cause of chronic hepatitis and end-stage liver disease. It is estimated that 71 million individuals have chronic HCV infections worldwide, and this is a serious public health concern [1]. Direct-acting antiviral (DAA) drugs are associated with a high cure rate for chronic hepatitis C [2], but the high cost is unaffordable for most patients [3]. In addition, the appearance of DAA-resistant HCV strains results in treatment failure [4–6], and DAA drugs do not prevent reinfection. As such, a vaccine against HCV remains urgently needed. HCV glycoproteins have not been shown to successfully induce a protective humoral or cellular immune response, most likely because of their high genetic diversity and weak immunogenicity.

Two main approaches have been explored for developing an HCV vaccine. HCV glycoproteins E1 and E2 have been used to elicit a protective humoral response and produce neutralizing antibodies (nAbs) [7, 8]. These nonstructural proteins have also been used to induce a broad cellular response [7]. In the phase I trial, however, E1/E2 with MF59 adjuvant only induced nAbs against HCV genotype 1a in a few subjects [9]. This may be partially attributed to the weak immunogenicity of HCV glycoproteins. In addition, the serum from poor responders exhibited antibody-dependent enhancement, which promoted HCV infection. It has been also reported that some nonneutralizing epitopes of the glycoproteins interfered with nAbs, thus facilitating HCV evasion [10, 11]. These intrinsic shortcomings are an obstacle to using E1/E2 as an immunogen for an HCV vaccine. On the other hand, a trivalent soluble E2 from HCV genotypes 1a, 1b, and 3a has been shown to induce a broad and synergistic humoral immune response in mice and nonhuman primates, indicating that E2 may be a preferred immunogen [12]. However, another study reported that E1 alone was able to induce a protective humoral response, while E2 failed to elicit nAbs [13].

The other approach is the development of cellular immunity-based vaccines. Swadling et al. demonstrated that adenoviral vectors expressing NS3-5 protein induced a CD8+ and CD4+ HCV-specific T cell response that was associated with viral control in humans [14]. However, a phase I/II clinical trial of this method that was completed in 2019 (NCT01436357) did not show superior efficacy to placebo.

HCV glycoprotein-derived peptides are generally considered to be less immunogenic and are rarely used to induce a protective humoral immune response. A peptide-induced humoral response seems unlikely to cover the hypervariable sequence of HCV glycoproteins to provide adequate protection. At least 2 decades ago, it was found that certain peptide-induced antisera can eliminate HCV infection [15–17]. However, most of these experiments were not conducted using the cell culture HCV (HCVcc) model [18, 19] because the model had not been established or widely used at that time.

This study is aimed at systematically evaluating the humoral response induced by conserved and nonconserved peptides within HCV genotype 1-6 glycoproteins using the HCVcc model.

## 2. Materials and Methods

### 2.1. Cells, Antibodies, and Peptides

Huh7.5 cells were a gift from Professor Charles Rice (Apath, L.L.C., and Rockefeller University, New York) and routinely maintained in Dulbecco's modified Eagle's medium (DMEM, Thermo Fisher, USA) supplemented with 10% fetal bovine serum (FBS) and 1% nonessential amino acids (aa). Anti-HCV core (SC-57800) and mouse anti-flavivirus (Cat. MAB10216) antibodies were purchased from Santa Cruz (USA) and Millipore (USA) companies, respectively.

**Figure 1:**
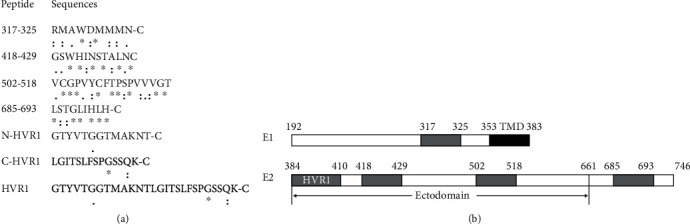
(a) Conservation analysis of segments within glycoproteins E1/E2. A total of 536 HCV sequences were submitted to Clustal Omega (http://www.ebi.ac.uk/Tools/msa/clustalo/) for alignment. An “∗” (asterisk) indicates positions that have a single, fully conserved residue. A “:” (colon) indicates conservation between groups of strongly similar properties, i.e., scoring > 0.5, in the Gonnet PAM 250 matrix. A “.” (period) indicates conservation between groups of weakly similar properties, scoring ≤ 0.5, in the Gonnet PAM 250 matrix. An extra cysteine residue was added to the C terminus of some peptides for conjugation with KLH. (b) Schematic diagram of the segments within glycoproteins E1/E2. TMD (black): transmembrane domain; HVR1: hypervariable region 1.

A total of 7 peptides were evaluated: four highly conserved peptides (aa positions according to H77 isolate YP_009506357.1) within E1 (aa317-325, N-RMAWDMMMN-C) and E2 (aa418-429, N-GSWHINSTALNC-C; aa502-518, N-VCGPVYCFTPSPVVVGT-C; and aa685-693, N-LSTGLIHLH-C) and 3 peptides within the HVR1 of E2 ([aa384-396] N-HVR1, N-GTYVTGGTMAKNT-C; [aa397-410] C-HVR1, N-LGITSLFSPGSSQK-C; and [aa384-410] HVR1, N-GTYVTGGTMAKNTLGITSLFSPGSSQK-C, aa positions according to CAB53095.1, genotype 1b). Peptides were synthesized by GL Biochem Company (China), and mass spectrometry analysis validated the purity of the peptide sequences to be >98%. An extra cysteine residue was added to the C terminus of some peptides to conjugate with keyhole limpet hemocyanin (KLH) protein using a Conjugation Kit (Figure 1(a); Cat. MBK1, Sigma, USA).

### 2.2. Sequence Alignment

For conservation analysis, a total of 536 HCV sequences were retrieved from the NCBI database and submitted to Clustal Omega (http://www.ebi.ac.uk/Tools/msa/clustalo/), as described in our previous study [20]. The sequences of the 7 synthesized peptides and TNcc(1a, AFX74877.1), J6/JFH1(2a, AEB71625.1), S52(3a, ADF97231.1), ED43(4a, CAA72338.1), SA13(5a, AC61696.1), and HK6a(6a, ACM69040.1) were also subjected to alignment with BioEdit 7.09 software (Figure S1).

### 2.3. Immunization of Mice

Antisera were generated by immunizing BALB/c mice with the 7 synthesized peptides. All animal studies were approved by the Institutional Animal Care and Use Committee of Guangzhou Eighth People's Hospital, and the animals were kept according to the institutional guidelines.

Fifty-four 6-week-old female BALB/c mice were purchased from GL Biochem Company (China) and divided into 9 groups (*n* = 6 for each group). Each group of mice was immunized with 1 peptide. One group of mice was immunized with KLH protein alone, and 1 group received no treatment; these groups were used as control groups. Briefly, 100 *μ*g of the peptide was conjugated with KLH protein at a molar ratio of 1000 : 1 using a Conjugation Kit (Cat. MBK1, Sigma, USA), according to the manufacturer's protocol. The KLH-conjugated peptide was then mixed with 100 *μ*L of complete Freund's adjuvant (CFA, Cat. F5881, Sigma, USA) for intraperitoneal inoculation at week 0. Fifty micrograms of KLH-conjugated peptide and 50 *μ*L of incomplete Freund's adjuvant (IFA, Cat. F5506, Sigma, USA) were mixed for reimmunization at weeks 2, 4, 5, 6, 7, and 8. KLH protein alone was also mixed with complete or incomplete Freund's adjuvant for inoculation as a control.

At week 9, all mice were euthanized with carbon dioxide. Antiserum titers were determined by an enzyme-linked immunosorbent assay (ELISA), and the IgG of pooled antisera was purified using Magne™ Protein G Beads (Promega, USA).

### 2.4. ELISA

Antiserum titers were measured using an indirect ELISA, as previously described [21]. Briefly, 96-well microplates preincubated with streptavidin were coated with biotinylated peptide (1 *μ*g/well), and then 2-fold dilutions of the mouse antisera were added to the wells. For each group, nonimmunized mouse serum was used as a negative control. After incubation at 37°C for 1 h and washing with PBS, horseradish peroxidase- (HRP-) conjugated goat anti-mouse IgG (Ray Antibody Biotech, China) was added, and the optical density (OD) values were measured at 450 nm using an ELISA reader (Tecan, USA). The endpoint titer was defined as the reciprocal of the highest dilution with a positive absorbance (OD > 0.2).

### 2.5. HCVcc Production and Neutralization Assay

HCVcc plasmids were a kind gift from Professor Jens Bukh (Hvidovre Hospital and University of Copenhagen, Denmark). HCVcc genotypes 1-6 including TNcc(1a) [22], JFH1-based J6 5′UTR-NS2 recombinant J6/JFH1(2a) [23], and isolate-specific 5′UTR-NS5A recombinants S52(3a), ED43(4a), SA13(5a), and HK6a(6a) [24] were generated and maintained as previously described, with some modifications [19]. Briefly, the cell culture supernatant was collected from 10 *μ*g of full-length HCV RNA-transfected Huh7.5 cells and was used to infect Huh7.5 cells grown in 100 mm dishes at a multiplicity of infection of 0.01. The infected cells were passaged at 3-day intervals. Approximately 2 weeks postinfection, viral supernatants were obtained and clarified by centrifugation and stored in aliquots at -80°C. The focus-forming units (FFU) of the viral stock was defined as HCV core-positive foci using an indirect immunofluorescence assay.

For the HCVcc neutralization assay, 6 × 10^3^ Huh7.5 cells were seeded onto a 96-well plate 1 day before infection. One hundred FFU of HCVcc stock was incubated with mouse antisera or IgG at 37°C for 1 h. The mixture was then incubated with Huh7.5 cells at 37°C for 6 h, and the medium was replaced with a fresh medium. At 72 h postinfection, HCV infection was evaluated in an indirect immunofluorescence assay. Each experiment was performed in duplicate.

For HCV indirect immunofluorescence assay, at 72 h postinfection, the cells in the 96-well plate were fixed with methanol at -20°C for 25 min and blocked with 1% bovine serum albumin (BSA) in PBS for 30 min, followed by incubation with a primary mouse anti-HCV core antibody [22] and secondary Alexa Fluor 488-conjugated goat anti-mouse antibody (Invitrogen, USA). The fluorescent foci were observed and enumerated on a standard fluorescence microscope (Olympus IX70).

### 2.6. Dengue Virus Neutralization Assay

Dengue virus (DV2; Thai strain 16681) was generously provided by Dr. Andrew Yue (National Health Research Institutes, Taiwan) [25]. Antisera were diluted 1 : 50, incubated with 10^4^ FFU Dengue virus for 1 h, and then added to 6 × 10^3^ Huh7.5 cells. Three days postinfection, DV2 infection was evaluated with a mouse anti-flavivirus group antigen monoclonal antibody in an indirect immunofluorescence assay, as described above.

### 2.7. Immunoprecipitation of Viral Particles and qRT-PCR

To reduce nonspecific binding, TNcc(1a) or ED43(4a) viral stock was incubated with 2 *μ*g of normal mouse IgG for 2 h at room temperature, followed incubation with 50 *μ*L of Dynabeads™ Protein G (Invitrogen, USA) for 30 min at room temperature. The viral stock (~106 IU) was mixed with 2 *μ*g of antiserum IgG for 2 h, followed by mixing with 50 *μ*L of Dynabeads™ Protein G for 30 min. The Dynabeads™ were washed 3 times with 200 *μ*L of cold PBS (5 min per wash) and then resuspended in 75 *μ*L of PBS. The HCV RNA was extracted from the immunoprecipitants with an E.Z.N.A.® Viral RNA Kit (Omega BIO-TEK, USA), according to the manufacturer's protocol, and then dissolved in 40 *μ*L nuclease-free water. The viral RNA was quantified by the PCR-fluorescent probe method with a commercial Diagnostic Kit for Quantification of HCV RNA (Cat. #DA-Z070, Da An Gene, China), according to the manufacturer's instructions [26].

### 2.8. Western Blotting

One hundred micrograms per milliliter of serum IgG and 400 FFU of HCVcc stock were incubated at 37°C for 1 h and then inoculated onto a 24-well plate preseeded with 3.6 × 10^4^ Huh7.5 cells 1 day before the experiment. Three days postinfection, the cells were washed with 500 *μ*L of PBS twice and digested with 100 *μ*L of Pierce IP Lysis Buffer (Cat. 87787) for 15 minutes on periodic ice mixing. The lysate was transferred to a microcentrifuge tube and centrifuged at 13,000 × *g* for 10 min at 4°C to pellet the cell debris. The protein concentration of the supernatant was measured using a Pierce BCA Protein Assay Kit (Cat. 23225).

Twenty micrograms of protein sample per lane was separated on an SDS-12% PAGE gel and transferred onto a polyvinylidene difluoride (PVDF) membrane (Cat. PVM020C-160, Pall). The membrane was blocked with 3% BSA in Tris-Buffered Saline and Tween 20 (TBST) for 0.5 h at room temperature and probed with a mouse anti-core antibody (1 : 200 dilution) with 1% BSA in TBST for 1 h at room temperature, or overnight at 4°C, followed by the addition of an HRP-goat anti-mouse secondary antibody (1 : 2000, Beijing Ray Antibody Biotech, China). The signal was detected with Pierce ECL Western Blotting Substrate (Cat. 32109) or High-Sig ECL Western Blotting Substrate (Cat. 180-501, Tanon Corp., China).

## 3. Results

### 3.1. Conservation Analysis of Peptides within HCV Glycoproteins

In a conservation analysis performed in our previous study [20], we manually downloaded 536 HCV sequences, including 85 genotype 1a, 272 genotype 1b, 2 genotype 1c, 20 genotype 2a, 82 genotype 2b, 5 genotype 2c, 37 genotype 3a, 1 genotype 3b, 10 genotype 4, 3 genotype 5, and 19 genotype 6 from the NCBI database. The alignment results showed that at least 4 segments (aa317-325, aa418-429, aa502-518, and aa685-693 within the HCV glycoproteins, aa positions according to H77 isolate YP_009506357.1) were highly conserved (Figures 1(a) and 1(b)). In contrast, the segment with the HVR1 of E2 was highly variable. Three peptides ([aa384-396] N-HVR1, N-GTYVTGGTMAKNT-C; [aa397-410] C-HVR1, N-LGITSLFSPGSSQK-C; and [aa384-410] HVR1, N-GTYVTGGTMAKNTLGITSLFSPGSSQK-C, aa positions according to CAB53095.1, genotype 1b) within this segment were also synthesized.

We also conducted an alignment of the sequences of peptide immunogens and HCVcc strains. As shown in Figure S1, aa317-325 and aa502-518 were absolutely conserved across the 6 genotypes, and the sequences of these 2 peptides were absolutely identical to the HCVcc tested. The sequence of peptide aa418-429 was absolutely identical to TNcc(1a) and S52(3a) and 91.7% (11/12) identical to J6/JFH1(2a), ED43(4a), SA13(5a), and HK6a(6a). The sequence of peptide aa685-693 was 88.9% (8/9) identical to J6/JFH1(2a) and absolutely identical to the other 5 genotypes. In contrast, segment HVR1 was highly variable across the 6 genotypes. Overall, it seemed that C-HVR1 was more conserved, as the similarities between peptide N-HVR1 and HCVcc were from 23.1% (3/13) to 61.5% (8/13), while the similarities between peptide C-HVR1 and HCVcc were from 50.0% (7/14) to 64.3% (9/14).

### 3.2. Protective Effect of Antisera

**Figure 2:**
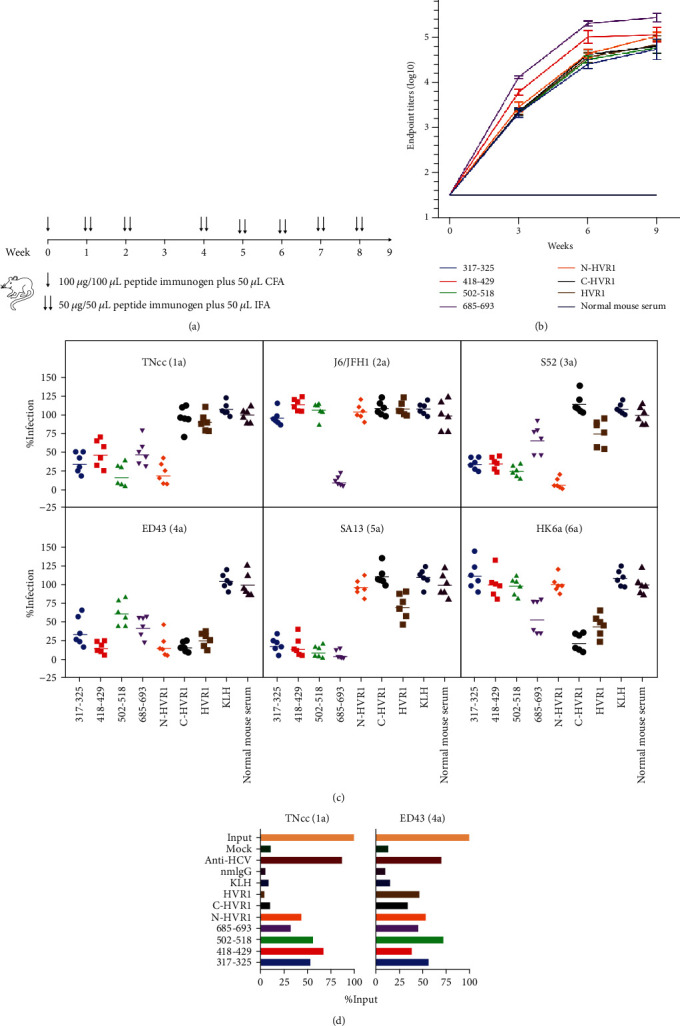
Humoral response induced by peptides. (a) Illustration of the immunization procedure. The peptide plus KLH protein, or PBS plus KLH protein, together with CFA or IFA, was inoculated into BALB/c mice at the indicated time points. (b) Kinetics of peptide-specific antibody titers of the antisera. The endpoint titers of antisera collected at weeks 0, 3, 6, and 9 were determined by ELISA. Sera from mice without immunization, defined as “normal mouse serum,” served as a baseline. The mean ± standard error of the mean (SEM) of each group is shown. (c) The antiserum of each mouse was diluted at 1 : 50, mixed with 100 FFU of HCVcc, and then added to naïve Huh7.5 cells. HCVcc without sera was added as a “mock” control. Seventy-two hours postinfection, the FFU was determined by an indirect immunofluorescence assay. The infection of “mock” was arbitrarily set to 100%, and the relative infection rates were calculated using GraphPad Prism 8 software. Each experiment was repeated in triplicate, and the representative data of 1 experiment is shown. The geometric mean of each group is displayed. (d) HCVcc TNcc(1a) or ED43(4a) stock (~106 IU as “input”) was precipitated with 2 *μ*g/mL IgG-coated Protein G Beads. Normal mouse IgG (nmIgG) and IgG purified from a convalescent hepatitis C patient (anti-HCV) were used as the negative and positive controls, respectively. HCVcc nonspecifically binding to Protein G Beads without an IgG coating was defined as a “mock.” HCV RNA of virus precipitated with beads was determined by qRT-PCR.

Seven peptides conjugated to KLH, or KLH alone was used to immunize BALB/c mice. The procedure is illustrated in Figure 2(a). The antibody titers of mouse antisera were determined at weeks 0, 3, 6, and 9 by ELISA (Figure 2(b)). The serum of a normal mouse without immunization across the whole experiment period served as a baseline. The antibody titers specific to the 7 peptides could be detected as early as 3 weeks postimmunization and continued to increase through week 6, then peaked (4.1-5.7 log10) at week 9, indicating that this immunization protocol can induce a potent and specific humoral response. We did not determine the antibody titer past week 9 because we believed that the titer at week 9 was sufficient for the following neutralization test.

To determine whether these antisera conferred protection against HCV infection, a 1 : 50 dilution of antisera was mixed with HCVcc of genotypes 1-6, and the infection rate was assessed by an indirect immunofluorescence assay (Figure 2(c)). As expected, normal mouse serum or antiserum induced by KLH alone had little effect on HCVcc infectivity. By contrast, both aa317-325 and aa418-429 antisera inhibited >50% of TNcc(1a), S52(3a), ED43(4a), and SA13(5a) infectivity. However, these antisera did not affect J6/JFH1(2a) or HK6a(6a) infectivity. The aa502-518 antisera efficiently neutralized 3 genotypes, including TNcc(1a), S52(3a), and SA13(5a), and mildly neutralized 39.5% of ED43(4a) but could not block infection of J6/JFH1(2a) or HK6a(6a). Antisera of aa685-693 stopped >50% of infections of TNcc(1a), J6/JFH1(2a), ED43(4a), and SA13(5a) and mildly inhibited S52(3a) and HK6a(6a). Interestingly, antisera of N-HVR1 potently prevented TNcc(1a), S52(3a), and ED43(4a) infections, but that of the other 3 genotypes (Table 1). Both C-HVR1 and full-length HVR1 antisera efficiently inhibited infections of ED43(4a) and HK6a(6a). To ensure that the protection of the antisera was HCV-specific, their neutralization activities against the Dengue virus, a member of the Flaviviridae family, were also evaluated using the same protocol. As shown in Table 1 and Figure S2, none of the antisera displayed an anti-Dengue effect.

To test whether the antisera could recognize HCV particles, we isolated the IgG of the pooled antisera of each group and determined their interactions with HCVcc in an immunoprecipitation assay. The precipitated HCV RNA was detected by qRT-PCR. Since all the antisera could neutralize the ED43(4a) strain, we tested whether the purified IgG recognized the HCVcc ED43(4a) particle first. Human IgG (anti-HCV) with broad neutralization activity purified from the serum of a convalescent hepatitis C patient served as a positive control. As shown in Figure 2(d), anti-HCV recognized 70.2% of HCVcc, while the IgG of normal mice and that induced by KLH only recognized <20%. A range of 34.3% to 74.2% of input HCVcc was immunoprecipitated by the IgG induced by the 7 peptides, indicating that the peptide immunogens indeed induced a genotype 4 HCV-specific humoral response. However, IgG antisera of C-HVR1 and HVR1 did not recognize TNcc(1a), and the HCV RNA levels were similar to those of the normal mouse IgG group, which may explain why they cannot affect TNcc(1a) infection. Taken together, these data highlight the protective effect of antisera induced by peptides within glycoproteins.

### 3.3. Protective Effect of Antiserum IgG

**Figure 3:**
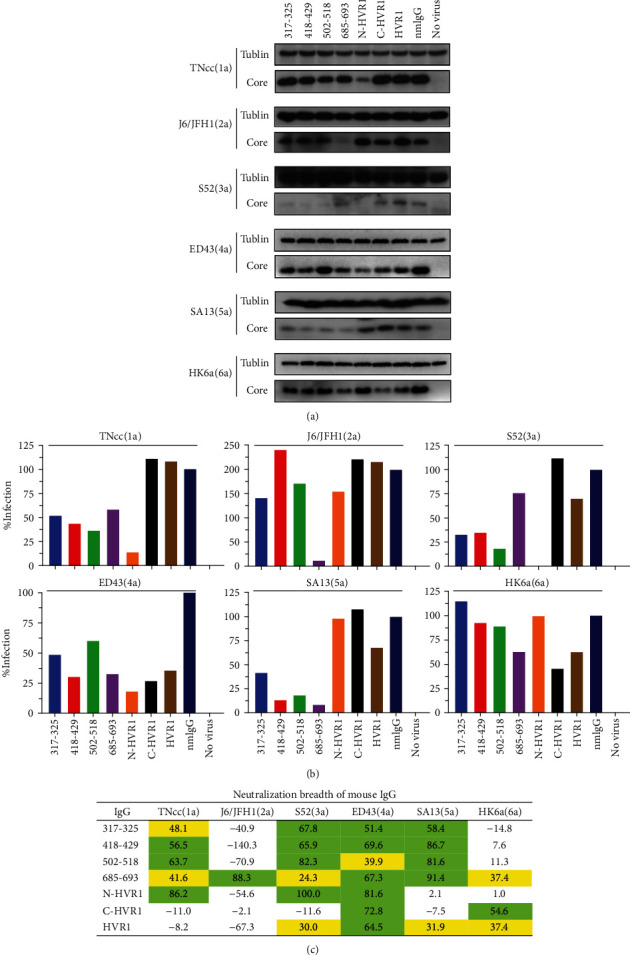
Neutralization activity of antiserum IgG. (a) IgG (100 *μ*g/mL) induced by each peptide was mixed with 400 FFU of HCVcc for 1 h and added to Huh7.5 cells preseeded in a 24-well plate. Seventy-two hours later, infectivity was evaluated by Western blotting. nmIgG: normal mouse IgG. (b) The density of each band was analyzed with ImageJ software, and the core/Tublin ratio was calculated. The infection of nmIgG was set to 100%, and the relative infection of each group was calculated with GraphPad Prism 8 software. (c) Neutralization breadth of mouse IgG (100 *μ*g/mL). Green, neutralization is >50%; yellow, neutralization is 20%-50%.

To exclude the possibility that other factors in antisera may contribute to the antiviral effect, the neutralization activities of IgG purified from antisera were determined. We mixed 100 *μ*g/mL of IgG and 400 FFU HCVcc stock and detected the infectivity in Huh7.5 cells using an anti-core monoclonal antibody for Western blotting. As shown in Figure 3(a), a band of ~17 kD could be detected in the normal mouse IgG group infected with HCVcc genotypes 1-6, but not in the “no virus” group, indicating that this antibody can effectively detect the HCV core antigen. After normalizing with the Tublin reference protein, the relative infection of each group compared to the “normal mouse IgG” group was determined (Figure 3(b)). The IgG of aa317-325, aa502-518, aa685-693, and N-HVR1 potently neutralized >50% of infection of the 3 genotypes, while aa418-429 IgG potently inhibited 4 genotypes (Figure 3(c)). For the J6/JFH1(2a) strain, only the IgG of aa685-693 exhibited remarkable neutralization activity. IgG of N-HVR1 exhibited the most potent inhibitory effect against genotypes 1, 3, and 4 but had little protective effect against the other 3 genotypes.

**Figure 4:**
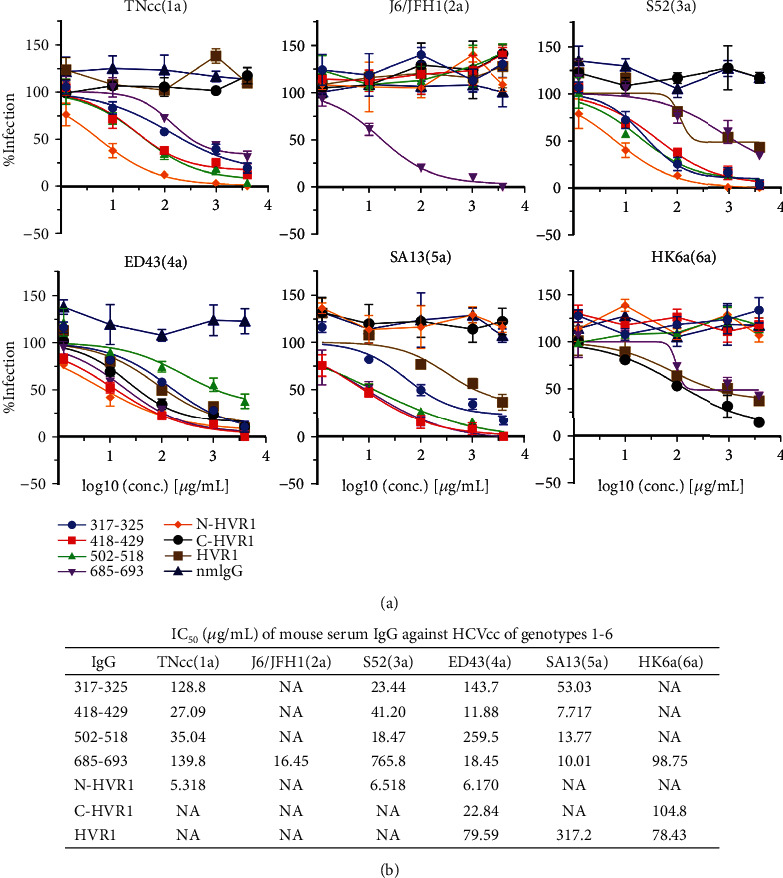
IC_50_ of mouse IgG. (a) Mouse IgG was serially diluted at 4000, 1000, 100, 10, and 1 *μ*g/mL, and neutralization activity was evaluated by a neutralization assay. Each experiment was performed in duplicate, and the mean ± SEM is shown. (b) The concentration of IgG inhibiting 50% of HCVcc infection was defined as the half-maximal inhibitory concentration (IC_50_) and calculated using a “variable slope” method with GraphPad Prism 8 software.

The neutralization activities of IgG were also evaluated at a greater concentration by an indirect immunofluorescence assay (Figure 4(a)). The results showed that only IgG of aa685-693 exhibited neutralization activity against HCVcc genotypes 1-6 (Figures 4(a) and 4(b)). However, even at 4000 *μ*g/mL, the highest concentration tested in this study, IgG of E2 aa685-693, could not completely prevent infections of TNcc(1a), S52(3a), and HK6a(6a). Consistent with the Western blotting result, IgG of N-HVR1 prevented TNcc(1a), S52(3a), and ED43(4a) infections, with an IC_50_ ranging from 5.318 to 6.518 *μ*g/mL.

## 4. Discussion

HCV glycoproteins are the main immunogens that induce a host humoral response during the natural course of infection, and most neutralizing epitopes within HCV glycoproteins are conformational [27, 28]. However, a vaccine against glycoproteins E1/E2 tested in chimpanzees exhibited effective protection against a homologous [29] but not a heterologous virus challenge [30]. In a phase I clinical trial of the E1/E2 vaccine, neutralizing activity against a homologous strain was detected only in about 25% of the volunteer sera [9]. These results indicate that optimization of the immunogen is required, and it was recently reported that altered glycosylation could enhance the immunogenicity of E2 and induce potent neutralizing antibodies [31].

Another strategy to induce a protective humoral response is the use of linear peptides as immunogens because linear epitopes have also been found within HCV glycoproteins [32]. The humoral response induced by linear peptides has been reported to be effective in preventing HCV infection by some genotypes [33, 34]. For example, the aa313-327 within E1 is highly conserved and immunodominant during HCV infection [35, 36]. Peptide aa315-323-induced antisera were shown to inhibit the binding and entry of genotype 4 HCV to HepG2 cells, and another vaccine using aa315-326 conferred protection against JFH1(2a) and ED43(4a) infections in hepatoma cells [33]. Two monoclonal antibodies targeting this epitope also prevented infections of HCV-pseudotyped particles (HCVpp) genotypes 1, 2, 4, 5, and 6 and HCVcc strains H77(1a), JFH1(2a), and J6/JFH1(2a). In this study, we found that a peptide aa317-325-induced humoral immune response could inhibit infections of HCVcc strains TNcc(1a), S52(3a), ED43(4a), and SA13(5a) but had little effect on infections of J6/JFH1(2a) and HK6a(6a) (Figure 3(c)). These data support that aa317-325 may serve as a vaccine immunogen for inducing a protective humoral immune response. Peptide aa502-518 is highly conserved and plays a crucial role in the association of HCV and host receptors [37]. Our results showed that aa502-518-induced antisera can efficiently neutralize 3 HCV genotypes, TNcc(1a), S52(3a), and SA13(5a). In contrast, Czarnota et al. recently reported that aa502-520 present on hepatitis B virus (HBV) small surface antigen virus-like particles could not induce neutralizing antibodies [38]. This discrepancy may be partially due to different immunogen coupling strategies; in our study, aa502-518 was conjugated to KLH protein. We found that aa418-429-induced antisera potently prevented infections of 4 genotypes, TNcc(1a), S52(3a), ED43(4a), and SA13(5a), but had little effect on J6/JFH1(2a) and HK6a(6a), while aa685-693-induced antisera strongly inhibited J6/JFH1(2a), ED43(4a), and SA13(5a) and mildly inhibited S52(3a) and HK6a(6a) (Figures 3(c) and 4(b)). Thus, a combination of these 2 peptide immunogens may induce neutralization of all HCV genotypes; however, this possibility requires further study.

The role of HVR1 within E2 with respect to vaccine development remains controversial. Induction of the HVR1 antibody is associated with an acute self-limited infection in which viral attachment is prevented [39, 40]. In addition, anti-HVR1 antiserum has been shown to prevent homogenous HCV infection *in vitro* and *in vivo* [15, 41]. In contrast, it has been reported that the HVR1 antibody interferes with nAbs [11], and thus, HVR1 should not be included in a vaccine [42]. However, Law et al. recently showed that the deletion of HVR1 did not result in better protection as compared with the wild-type E1/E2 immunogen [43]. In this study, the antiserum of the full-length HVR1 of genotype 1b potently inhibited ED43(4a) and mildly inhibited S52(3a), SA13(5a), and HK6a(6a) infections but did not influence TNcc(1a) strain infectivity. In fact, despite the extremely high sequence diversity of HVR1, it has been reported that there is significant cross-reactivity between its variants [44, 45]. This may explain why the HVR1 of 1 genotype of HCV can elicit nAbs against other genotypes. The neutralization epitopes in HVR1 are generally considered to be within the C terminus, and monoclonal nAbs against this segment have been reported [46–48]. We found that C-HVR1 elicited nAbs against HCVcc of 2 genotypes, ED43(4a) and HK6a(6a). Unexpectedly, the N-HVR1 of genotype 1b elicited the most potent nAbs against 3 genotypes, 1, 3, and 4 (Figure 3(a)), while it had little effect on other genotypes. Indeed, Mosa et al. recently reported that patient-derived HVR1 peptides induced cross-genotype neutralization [49]. Since nAbs targeting the C terminus of HVR1 usually have stringent isolate specificity [50], there is a possibility that N-HVR1 peptide can induce a protective humoral immune response against various genotypes. Whether N-HVR1 peptide will have the same effect on genotypes other than 1b needs to be further explored.

Conservation variance may not account for the distinct neutralization activities of the antibodies induced by peptide immunogens. For example, the sequences of peptides aa317-325 and aa502-518 were absolutely identical to the HCVcc 6 genotypes tested in this study; however, IgG induced by these 2 peptides had little effect on J6/JFH1(2a) and HK6a(6a) but neutralized the other 4 genotypes. The sequence of aa418-429 was 91.7% (11/12) identical to J6/JFH1(2a), ED43(4a), SA13(5a), and HK6a(6a), but the IgG induced by aa418-429 only efficiently neutralized 2 of the 4 genotypes, ED43(4a) and SA13(5a). Peptide aa685-693 was the only immunogen that induced potent neutralization activity against J6/JFH1(2a) in this study. However, the humoral response induced by aa685-693 did not neutralize the other 5 HCVcc genotypes examined, although their sequences were absolutely identical. N-HVR1 is less identical to the HCVcc 6 genotypes compared to C-HVR1 (similarity 23.1% to 61.5% vs. 50.0% to 64.3%, Figure S1); however, IgG induced by N-HVR1 potently neutralized 3 genotypes while IgG induced by C-HVR1 only neutralized 2 genotypes (Figure 3). These findings suggest that the neutralization activity against heterologous strains induced by N-HVR1 is less likely to be dependent on sequence similarity. Recently, another group also reported that the sequence similarity of HVR1 was not associated with its cross-neutralization [49].

In this study, we determined the neutralization activities of IgG directly purified from antisera and confirmed the protective humoral responses elicited by peptide immunogens. Nevertheless, we did not determine the peptide related to cellular immune response, which also plays a central role in controlling HCV infection [3, 51, 52]. A comprehensive evaluation of both the cellular and humoral responses induced by the peptides in nonhuman primate models would undoubtedly provide a more complete and better understanding of peptide immunology and facilitate vaccine development.

## 5. Conclusions

Conserved peptides aa317-325, aa418-429, aa502-518, and aa685-693 efficiently induced antibodies with potent neutralizing activity against 3 or 4 HCV genotypes. The peptide aa685-693 within the C terminus of E2 elicited antibodies that achieved more than 50% neutralization of genotypes 2, 4, and 5 and less than 50% for genotypes 1, 3, and 6. Peptide N-HVR1 of E2 elicited antibodies with potent neutralization activities against 3 genotypes, TNcc(1a), S52(3a), and ED43(4a). These findings suggest that peptides within conserved and variable regions of envelope proteins could serve as immunogen candidates for an HCV vaccine.

## Figures and Tables

**Table 1:** NT_50_ of mouse antisera against HCVcc of genotypes 1-6 and Dengue virus.

Antisera	HCVcc						DV2
	TNcc(1a)	J6/JFH1(2a)	S52(3a)	ED43(4a)	SA13(5a)	HK6a(6a)	
317-325	50	<25	50	50	100	<25	<25
418-429	50	<25	50	200	100	<25	<25
502-518	200	<25	100	25	200	<25	<25
685-693	50	800	25	50	800	25	<25
N-HVR1	200	<25	800	200	<25	<25	<25
C-HVR1	<25	<25	<25	200	<25	100	<25
HVR1	<25	<25	25	100	25	50	<25
KLH	<25	<25	<25	<25	<25	<25	<25
NMS	<25	<25	<25	<25	<25	<25	<25

Mouse antisera of each group were pooled for neutralization tests. The NT_50_ was defined as the highest dilution of sera able to neutralize 50% of infection. The data are representative results from 3 independent experiments. NMS: normal mouse sera; DV2: Dengue virus Thai strain 16681.

## Data Availability

The research data used to support the findings of this study are available from the corresponding author upon request.

## Supplementary Materials

Supplementary Materials Figure S1: (A) alignment of peptide sequences; (B) similarities between the sequences of peptides and 6 HCVcc genotypes. Figure S2: the antisera of each group were diluted at 1 : 50 and incubated with 10^4^ FFU of Dengue virus (DV2; Thai strain 16681) for 1 h, followed by the addition to naïve Huh7.5 cells. At 72 h postinfection, the virus foci were determined by an indirect immunofluorescence assay.
