# Supplementary figures and images for: Keyhole Limpet Hemocyanin-Conjugated Peptides from Hepatitis C Virus Glycoproteins Elicit Neutralizing Antibodies in BALB/c Mice
Source: J Immunol Res. 2021 Jan 16;2021:3108157. doi: 10.1155/2021/3108157 (PMC7834783; doi:10.1155/2021/3108157)

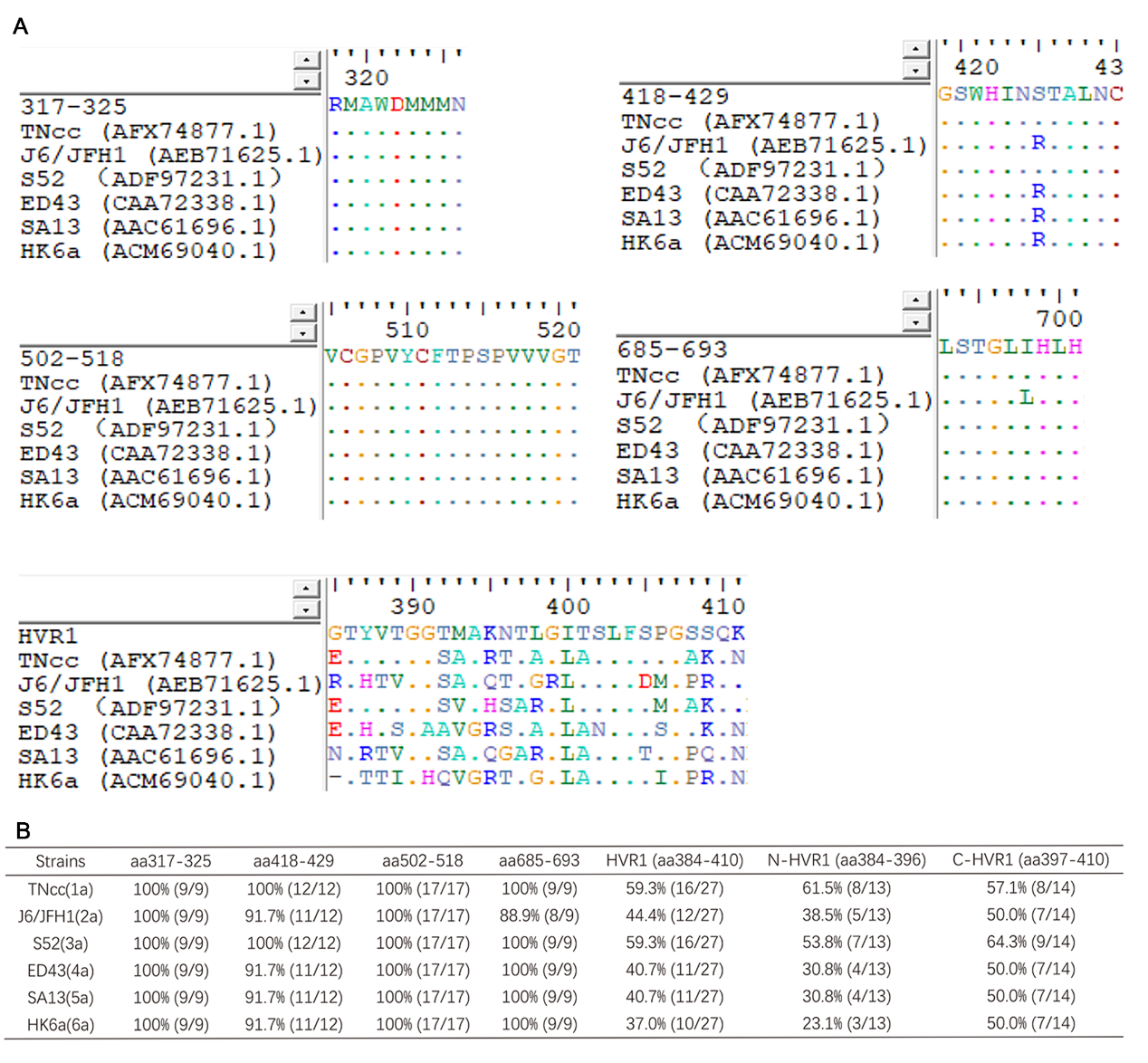

Supplement: Supplementary Materials — Figure S1: (A) alignment of peptide sequences; (B) similarities between the sequences of peptides and 6 HCVcc genotypes. Figure S2: the antisera of each group were diluted at 1 : 50 and incubated with 104 FFU of Dengue virus (DV2; Thai strain 16681) for 1 h, followed by the addition to naïve Huh7.5 cells. At 72 h postinfection, the virus foci were determined by an indirect immunofluorescence assay. [file 3108157.f1.zip › 3108157.f1.docx]

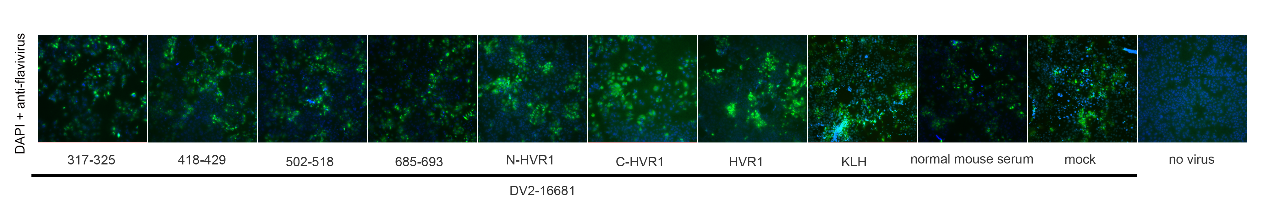

Supplement: Supplementary Materials — Figure S1: (A) alignment of peptide sequences; (B) similarities between the sequences of peptides and 6 HCVcc genotypes. Figure S2: the antisera of each group were diluted at 1 : 50 and incubated with 104 FFU of Dengue virus (DV2; Thai strain 16681) for 1 h, followed by the addition to naïve Huh7.5 cells. At 72 h postinfection, the virus foci were determined by an indirect immunofluorescence assay. [file 3108157.f1.zip › 3108157.f2.docx]
